# Chronic Pain Hurts the Brain: The Pain Physician's Perspective

**DOI:** 10.1155/2020/3786562

**Published:** 2020-03-21

**Authors:** Gergely Feher, Delia Szok, Joel Rodríguez-Saldaña, Ferenc Nagy

**Affiliations:** ^1^University of Pécs, Pécs, Hungary; ^2^University of Szeged, Szeged, Hungary; ^3^Multidisciplinary Diabetes Center, Mexico City, Mexico; ^4^Somogy County Kaposi Mór Teaching Hospital, Kaposvár, Hungary

Chronic pain is a devastating disease which can affect 10-20% of the whole population [1]. It is one of the prominent cases of disability worldwide. For example, lower back and neck pain is the leading cause of disability and migraine is ranked as the third in the jobholder population [2].

Chronic pain has a significant impact on both individuals and society. The costs of chronic musculoskeletal pain can reach 0,5% of the whole GDP based on a study from Chile, while the economic cost of migraine can be as high as £835 million annually in the UK [3, 4].

Despite of intensive pain research in the last 3 decades, patient management is still a challenge for physicians and patients may have to take long journeys until finding a qualified specialist to receive appropriate treatment. For example, a recent study showed that 40 patients suffering from intractable chronic pain consulted the following number of practitioners: general physicians 461, pain specialists 172, psychologist/psychiatrists 104, and universities 23 [5].

Although advances have been made for the treatment of chronic pain, it remains inadequately controlled for many people. The vast majority of physicians are focusing only on the casual background of pain (e.g., injury or arthropathy) leading to unnecessary imaging and uncontrolled prescription conventional painkillers or opioids [1].

Conventional analgesic drugs (NSAIDS) are minimally effective and overused in the management of chronic pain, leading to serious adverse effects and complications such as heart attack, kidney failure, and gastrointestinal bleeding. According to FDA recommendations, NSAIDs should be administered at the lowest effective dose for the shortest duration consistent with individual patient treatment goals, and consequently, NSAIDs have a very limited use in the management of chronic pain. However, a significant increase could be found in the prescription of these drugs worldwide in the absence of supporting evidence [6].

If properly selected, opioids can be efficacious but are also associated with addiction. The overuse of these agents has led to the opioid epidemic in the USA, in which in 2015 nearly 33,000 deaths were attributable to overdose with licit and illicit opioids [7].

Although clinical phenotypes of different pain syndromes are variable, they are linked through neuropsychiatric complications that include mood disorders, persistent fatigue, cognitive dysfunction, headache, irritable bowel syndrome, and insomnia [1].

Cognitive, psychosocial, and emotional factors have a critically important influence on pain perception, due to the connectivity of brain regions controlling pain perception, attention or expectation, and emotional states. Imaging studies have confirmed altered activity of afferent and descending pain pathways, as well as atrophy of different pain perception regions of the brain, which can result in psychiatric symptoms.

The introduction of the neurophysiological model of pain during the past decade stimulated the development of more therapeutically effective and cost-effective interdisciplinary chronic pain management programs including pharmacological and cognitive therapies.

Chronic pain often has neuropathic components. This kind of pain originates from injury to the peripheral or central nervous system resulting in maladaptive changes in neurons along the nociceptive pathway [8]. In addition to diabetic neuropathy and several common neuropathic pain syndromes, there is limited evidence regarding the treatment of chronic pain; therapeutic strategies are mainly based on the most likely mechanism(s) of pain, instead on therapies based focusing on the cause of pain. This paradigm however may be difficult to implement in clinical practice [1, 8].

In this issue focusing on chronic pain, Szok and her coworkers gathered literature-based evidence in the management of neuropathic pain, which may also help to make therapeutic decisions in the therapy of patients suffering from intractable or chronic pain as there is a significant overlap between these entities. The management of chronic pain, apart from the most common syndromes, is still a challenge for clinicians, and we also struggle with the lack of high-quality evidence (for example, orofacial pain). This extensive review integrates the latest International Association for the Study of Pain (IASP) classification of chronic pain with the International Classification of Diseases (ICD-11). Both pharmacological and nonpharmacological interventions are discussed with the level of supporting evidence which may help clinicians to guide treatment.

Pal et al. presented a cross-sectional, single-institution, prospective study including a cohort of patients investigated with small fiber neuropathy (SFN) between the years of 2012 and 2018 in a tertiary center. SFN is a disabling and often unrecognized neuropathic pain syndrome with great impact on quality of life. Treatment is often difficult due to the heterogeneous epidemiology and the lack of randomized studies. Their work guides us in the diagnostic workup of SFN both in idiopathic and secondary forms. Based on their results—in parallel with a limited number of previous studies—all recommended tests have to be done to exclude the potentially treatable forms; otherwise, only symptomatic therapy is available for patients.

Halicka and her coworkers highlight a poorly understood neuropathic condition, chronic regional pain syndrome (CRPS), focusing on neuropsychological changes in their in-depth review. CRPS has been described as a devastating pain syndrome associated with autonomic dysfunction, swelling, dystrophic skin changes, stiffness, functional impairment, and eventual atrophy. This review covers the complex neuropsychological changes associated to CRPS that include distortions in body representation, deficits in lateralised spatial cognition, and nonspatially lateralised higher cognitive functions, possibly related to the disruption of parietal cortical networks sharing similarities with structural brain lesions or chronic pain syndromes. These cognitive changes help to better understand brain networks involved in pain processing and can be targets of future nonpharmacological interventions of both CRPS and other chronic pain syndromes.

Abandoning neuropathic pain syndromes, Bank and his workgroup showed the possible association of migraine and cardiovascular risk. As several epidemiological and prospective studies showed a link between migraine (especially migraine with aura) and cardio- and cerebrovascular events, they conducted a modified Framingham score-based evaluation of vascular event-free middle-aged migraineurs referred to their headache clinic. Their article draws attention to the higher cardiovascular risk of middle-aged migraineurs and highlights the deficiency of primary prevention as most migraineurs had higher cardiovascular risk comparing to nonmigraineur populations, and the vast majority of moderate- and high/very high-risk patients did not reach the recommended metabolic targets. Their article describes the cardiovascular aspects of migraine and based on their results requires a holistic approach instead of focusing only on pain and pain relief, underlying the complexity of this common pain syndrome.

*Gergely Feher* *Delia Szok* *Joel Rodríguez-Saldaña* *Ferenc Nagy*
